# The impact of hyperkalemia on ICU admission and mortality: a retrospective study of Chinese emergency department data

**DOI:** 10.1186/s12873-024-01011-z

**Published:** 2024-06-01

**Authors:** Jian Sun, Qingyuan Liu, Samuel Seery, Lu Sun, Yuan Yuan, Wenwen Wang, Yan Wang, Ziwei Cui, Yueguo Wang, Yulan Wang, Jiashan Zhu, Mengping Zhang, Yinglei Lai, Kui Jin

**Affiliations:** 1 Department of Emergency Medicine, Division of Life Sciences and Medicine, The First Affiliated Hospital of USTC, University of Science and Technology of China, Hefei, Anhui 230001 China; 2 School of Mathematics and Physics, Anhui Jianzhu University, Hefei, Anhui 230009 China; 3 School of Mathematical Sciences, University of Science and Technology of China, 96 Jinzhai Road, Hefei, Anhui 230026 China; 4 Faculty of Health and Medicine, Division of Health Research, Lancaster University, Lancaster, LA1 4YW UK

**Keywords:** Hyperkalemia, Emergency room, Mortality risk, Disease severity

## Abstract

**Objective:**

This study assesses the influence of hyperkalemia on both disease severity and the risk of mortality among patients admitted to the emergency room.

**Methods:**

This retrospective observational study utilized data from the Chinese Emergency Triage Assessment and Treatment database (CETAT, version 2.0), which was designed to evaluate and optimize management strategies for emergency room (ER) patients. Patients were systematically categorized based on serum potassium levels. Relationships between serum potassium levels, risk of mortality, and the severity of illness were then analyzed using multifactorial logistic regression and through Receiver Operating Characteristic (ROC) analysis. The effectiveness of various treatments at lowering potassium levels was also investigated.

**Results:**

12,799 emergency patients were enrolled, of whom 20.1% (*n* = 2,577) were hypokalemic and 2.98% (*n* = 381) were hyperkalemic. Among hyperkalemic patients, the leading reasons for visiting the ER were altered consciousness 23.88% (*n* = 91), cardiovascular symptoms 22.31% (*n* = 85), and gastrointestinal symptoms 20.47% (*n* = 78). Comparative analysis with patients exhibiting normal potassium levels revealed hyperkalemia as an independent factor associated with mortality in the ER. Mortality risk appears to positively correlate with increasing potassium levels, reaching peaks when blood potassium levels ranged between 6.5 and 7.0. Hyperkalemia emerged as a strong predictor of death in the ER, with an Area Under the Curve (AUC) of 0.89. The most frequently prescribed treatment for hyperkalemia patients was diuretics (57.32%, *n* = 188), followed by intravenous sodium bicarbonate (50.91%, *n* = 167), IV calcium (37.2%, *n* = 122), insulin combined with high glucose (27.74%, *n* = 91), and Continuous Renal Replacement Therapy (CRRT) for 19.82% (*n* = 65). Among these, CRRT appeared to be the most efficacious at reducing potassium levels. Diuretics appeared relatively ineffective, while high-glucose insulin, sodium bicarbonate, and calcium preparations having no significant effect on the rate of potassium decline.

**Conclusion:**

Hyperkalemia is common in emergency situations, especially among patients with altered consciousness. There is a strong positive correlation between the severity of hyperkalemia and mortality risk. CRRT appears to be the most effective potassium reducting strategy, while the use of diuretics should be approached with caution.

## Background

Hyperkalemia is a frequently encountered electrolyte imbalance in clinical settings [1], Which is traditionally defined as a serum potassium level > 5.5 mmol/l [2]. This condition can give rise to various complications, notably cardiac arrhythmias, wherein elevated potassium disrupt the heart’s electrical impulses, potentially causing in life-threatening arrhythmias like ventricular tachycardia or fibrillation. Patients with hyperkalemia may also manifest symptoms such as muscle weakness, fatigue, and, in severe cases, paralysis. Hence, early diagnosis of hyperkalemia is of critical importance for patients visiting emergency rooms (ERs). However, the incidence of hyperkalemia in ERs varies across studies suggesting an approximate range of 1.7–2.6% [3]. Patients particularly susceptible to hyperkalemia are those with chronic kidney disease, diabetes, cardiovascular disease, and those on medications such as angiotensin-converting enzyme inhibitors and/or aldosterone antagonists [2]. Given the prevalence of hyperkalaemia among these at-risk populations, a comprehensive examination of the reasons for ER visits, patient management, and the effect of this becomes imperative.

Failure to promptly address elevated potassium levels can lead to severe consequences, such as cardiac arrest and arrhythmias, significantly increasing the risk of patient mortality [4, 5]. Research highlights a correlation between serum potassium levels in hyperkalemic patients and the occurrence of cardiovascular complications, as well as a 28-day mortality risk among hospitalized patients [6]. Immediate interventions are imperative in cases of severe hyperkalemia (serum potassium > 6.0-6.5mmol/L), as this condition poses a potential life-threatening risk [7]. The judicious selection and timely administration of effective treatments to reduce potassium levels are critical to managing emergency cases in involving hyperkalemia. However, current treatment modalities for hyperkalemia exhibit significant variability. In a study by Singer et al. [8] calcium emerged as the most common choice for emergency hyperkalemia patients in Suffolk County, Massachusetts. By contrast, Peacock et al. [9] found in the ER insulin/glucose therapy was the most commonly employed, either alone or in combination with other treatments, constituting 64% of cases. This finding aligns with a study by Wu et al. [10] conducted in China. However, despite these insights, the most effective intervention for hyperkalemia treatment in ERs remains unclear.

In order to comprehensively explore hyperkalaemia epidemiology and to understand the influence of this condition in relation to outcomes, this study relies on the robust and extensive data available within the Chinese Emergency Triage, Assessment, and Treatment (CETAT) database. The dataset spans from January 2020 to July 2021 and involves a diverse range of cases treated in emergency resuscitation rooms, details of the data set are published elsewhere [11]. Leveraging this rich source of information, the study aims to equip emergency physicians with nuanced insights into the varying severity levels of hyperkalemia and the associated risks of mortality across a spectrum of serum potassium concentrations. The methodological approach hinges on this data to offer a foundation for timely and informed decision-making, ultimately contributing to the optimization of treatment strategies for patients with hyperkalemia.

## Methods

## Participants

The data utilized in this study was abstracted from the multicenter emergency triage database of the Chinese Emergency Specialty Medical Association (CETAT), version 2.0. This database integrates information from patients admitted to the emergency rooms of **eight** tertiary teaching hospitals nationwide (Peking Union Medical College Hospital, The First Affiliated Hospital of University of Science and Technology of China, The Cardio-Cerebrovascular Hospital affiliated with the University of Science and Technology of China, Suqian People’s Hospital, Wuhan University People’s Hospital, Huai’an Second People’s Hospital, Hefei No. 2 People’s Hospital, and Subei People’s Hospital), amounting to over 80,000 records. The University of Science and Technology of China’s (USTC) School of Mathematics supervise CETAT data quality. All data undergo privacy protection processes, with personal identifiers systematically removed. Data were abstracted from January 2020 to July 2021, focusing on patients admitted to the resuscitation room at the First Affiliated Hospital of USTC and The Cardio-Cerebrovascular Hospital affiliated with the University of Science and Technology of China (Data are available upon request from the corresponding author).

This research strictly adhered to medical ethical standards and has obtained approval from the Ethics Committee of the First Affiliated Hospital of USTC (USTC reference no.22KY079). Given the retrospective nature of this study and the absence of any clinical intervention, the hospital ethics committee waived the requirement for patient-informed consent forms.

### Eligibility criteria

#### Inclusion

- Age ≥ 18 years old;
- The patient, upon admission, must have available laboratory test results for blood potassium, and complete necessary data.

#### Exclusion

- Incomplete data or lost to follow-up.
- Patients with hyperkalemia who did not undergo a recheck their blood potassium levels within 24 h;
- Individuals who requested restrictive treatment and did not adhere to the prescribed medical treatments by the doctor.

### Definition and outcomes

Patients were classified based on their initial biochemical serum potassium ion concentrations upon admission for emergency care measured using ion-selective electrode method. Hyperkalemia is defined according to the international standards [12], as a serum potassium concentration exceeding 5.5 mmol/L (1 mmol/L = 1 meq/L). Patients with hyperkalaemia were further classified as either: stage I (Mild hyperkalemia,5.5< [K^+^] ≤ 6mmol/L), stage II (Moderate hyperkalemia, 6< [K^+^] ≤ 6.5mmol/L), stage III (Severe hyperkalemia,6.5< [K^+^] ≤ 7mmol/L) or stage IV (Extreme hyperkalemia, [K^+^] > 7mmol/L).

Patients were allocated to one of nine categories based on their primary complaints at the time of presentation. These included cardiovascular symptoms, consciousness disorders, gastrointestinal symptoms, respiratory symptoms, urinary system symptoms, endocrine symptoms, trauma, cardiopulmonary arrest, among others. Categorization relied on the predominant complaints observed during patient presentations and was determined by three emergency doctors who were not blinded to the research plan. In cases where a patient had two or more complaints, classification was based on the primary reason for presentation.

The potassium reduction rate (∆k/k1) was defined as the difference (∆k) between the initial blood potassium level (k1) at hospital admission and the lowest blood potassium level (k2) reviewed within 24 h as a percentage of the initial blood potassium level (k1) upon entry. If ∆k/k1 ≥ 25%, it indicates a faster potassium reduction rate, and vice versa.

### Data Collection

After obtaining hospital ethical board approval (USTC reference No.22KY079.), we obtained data from the CETAT database, which includes all patients who were admitted to the ER at the first affiliated hospital of USTC from 2020 to 2021. Information containing demographic data, diagnoses, chief complaint at presentation, laboratory results, and various test elements such as reports, triage summaries as well as physiology information, which includes BP, respiration rate, temperature, heart rate, oxygen et al. were collected. Once diagnosed as hyperkalemia the following treatments.

Patients diagnosed with hyperkalemia had their subsequent management measures within 24 h after enrollment collected by four clinical physicians, primarily including the following interventions: administration of calcium gluconate or calcium chloride to stabilize cardiac membranes, insulin and glucose to facilitate potassium entry into cells, sodium bicarbonate for cases of metabolic acidosis, beta-2 agonists to stimulate potassium uptake, and diuretics to enhance potassium excretion.

### Outcomes

The primary outcome is mortality in the emergency room following admission. The secondary outcome is progression of patient’s condition to critically ill status, define as the necessity for intensive care unit (ICU) admission, categorized as the high-risk (HR) group. The third study endpoint investigates whether the potassium reduction rate for the mentioned five potassium-lowering measures exceeds 25%.

### Statistical analysis

Statistical analysis was performed by personnel not informed of the research plan and independently by two statistical experts who cross-checked the related results. For continuous variables, Wilcoxon’s rank-sum test compares differences between groups. The Chi-squared test or Fisher’s exact test were used to compare inter-group differences for categorical variables. Odds ratios (OR) were used compares in-hospital mortality rates between the hyperkalemia and the normal potassium level groups. The influence of different serum potassium levels in hyperkalemia on patients’ severity and mortality risk is assessed using binary logistic regression with other factors as independent variables. A p-value of < 0.05 was taken as the threshold for statistical significance.

## Results

### Baseline characteristics and exposures

**Fig. 1:**
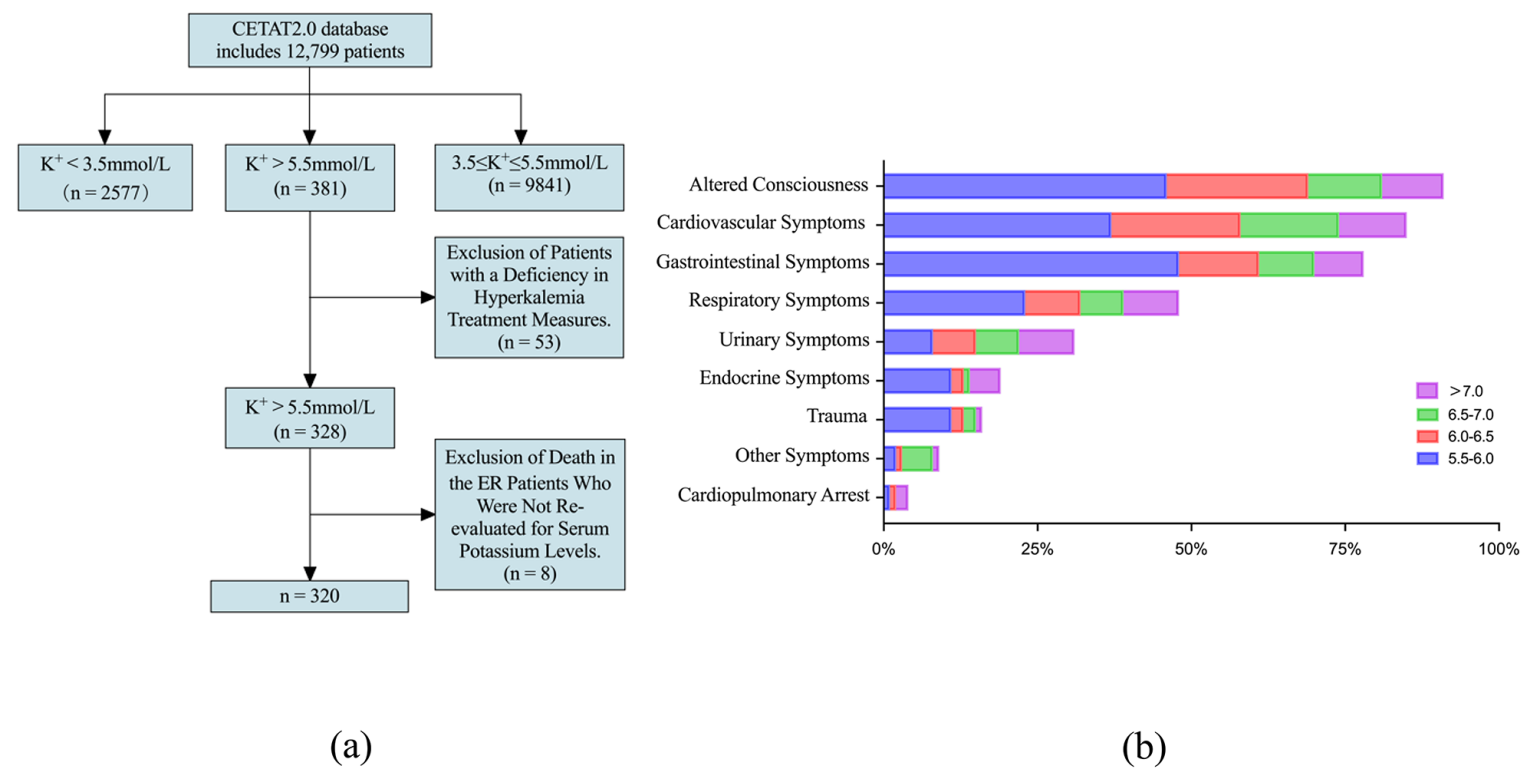
Participant selection process(**a**), and Percentage Of Patients With Hyperkalemia In The Emergency Room With Related Symtoms (**b**)

A total of 12,799 patients were included, among whom 2.98% (*n* = 381) presented with hyperkalemia. Notably, data related to potassium-lowering treatments were unavailable for 53 of these patients, eight of whom succumbed upon admission to the ER without a secondary potassium check. Consequently, the final study group comprises hyperkalaemia 320 patients with hyperkalaemia (see Fig. 1(a) for further details). In 381 patients with hyperkalemia, stage IV hyperkalemia was observed in 0.44% (*n* = 56), stage III hyperkalemia in 0.46% (*n* = 59), stage II hyperkalemia in 0.66% (*n* = 79), and stage I hyperkalemia in 1.46% (*n* = 187). The three most common complaints among hyperkalemia patients at presentation were altered consciousness at 23.88% (*n* = 91), cardiovascular symptoms at 22.31% (*n* = 85), and gastrointestinal symptoms at 20.47% (*n* = 78), see Fig. 1(b) for further details.

Patients with hyperkalemia generally present with more severe conditions compared to those without hyperkalemia. In general, individuals with hyperkalemia exhibited lower body temperatures, blood pressures, blood oxygen levels, hemoglobin concentrations, platelet counts, albumin levels and so forth. Conversely, they displayed higher SOFA and NEWs scores, as well as increased age, heart rate, white blood cell count, percentage of neutrophils, creatinine, phosphorus, and anion gap analysis (see Table 1 for details). Multifactorial logistic regression analysis indicated that age, body temperature, sodium ion concentration, hematocrit (red blood cell ratio), hemoglobin concentration, creatinine, PT (prothrombin time), and carbon dioxide binding capacity are independent factors associated with hyperkalemia. See Table 2 for further details.

**Table 1:** Demographics and clinical information with measures of significance

Variables		Normal Potassium (*N* = 9,841)	Hyperkalemia (*N* = 381)	*p*
Gender	Male	6,396 (65.0%)	235 (61.7%)	0.18
Age (years)		64.0 (52.0,74.0)	69.0 (54.0,79.0)	< 0.01
Admission method	Contacted emergency responders	4,763 (48.4%)	211 (55.4%)	< 0.01
Level of consciousness	Clearer	7,155 (72.7%)	247 (64.8%)	< 0.01
	Blurred	1,253 (12.7%)	49 (12.9%)	
	Coma	1,433 (14.6%)	85 (22.3%)	
Temperature(℃)		36.5 (36.3,36.6)	36.4 (36.2, 36.6)	< 0.01
Heart rates(beats/min)		82.0 (72.0, 98.0)	90.0 (75.0,108.0)	< 0.01
Respiratory rate(breaths/min)		20.0 (20.0,21.0)	20.0 (20.0,22.0)	< 0.01
SBP(mmHg)		139.0 (119.0,161.0)	126.0 (106.0,153.0)	< 0.01
DBP(mmHg)		82.0 (71.0, 95.0)	72.0 (58.0,90.0)	< 0.01
Spo2(%)		96.0 (95.0,98.0)	95.0 (89.0,98.0)	< 0.01
WBC(×10^9^/L)		9.2 (6.8,12.5)	10.9 (7.8,16.0)	< 0.01
NEUT%		80.3 (70.4, 87.5)	84.8 (77.7, 89.5)	< 0.01
Hematocrit		38.5 (33.1, 42.7)	33.4 (26.2, 41.9)	< 0.01
Hemoglobin (g/L)		129.0 (110.0, 143.0)	110.0 (84.0,136.0)	< 0.01
PLT(×10^9^/L)		181.0 (140.0,229.0)	180.0 (131.0,254.0)	0.55
ALT (U/L)		21.0 (14.0,34.9)	22.4 (13.8,50.8)	0.03
AST (U/L)		25.0 (19.0,39.0)	33.0 (19.0,77.7)	< 0.01
Albumin(g/L)		40.4 (36.3, 43.7)	37.4 (32.7,41.8)	< 0.01
TBIL(umol/L)		12.3 (8.6, 18.1)	11.8 (6.5,21.9)	0. 11
Creatinine(umol/L)		70.5 (56.0,92.0)	214.2 (121.0,522.0)	< 0.01
GFR(ml/min/m^2^)		98.9	28.2	< 0.01
CO2CP(mmol/L)		23.4 (20.9, 25.8)	15.6 (10.0,20.5)	< 0.01
Sodium(mmol/L)		139.0 (136.4,141.0)	135.0 (131.0, 140.0)	< 0.01
Phosphorus(mmol/L)		1.1 (0.9,1.3)	1.8 (1.4,2.7)	< 0.01
Anion Gap		13.0 (9.6,16.6)	20.6 (15.5,28.0)	< 0.01
Osmolality(mmol/L)		282.4 (277.4,288.0)	294.1 (283.7, 311.3)	< 0.01
Glucose(mmol/L)		7.2 (6.0,9.2)	8.3 (6.3, 13.7)	< 0.01
PT(secs.)		13.2 (12.5,14.2)	14.6 (13.0,17.0)	< 0.01
APTT(secs.)		34.1 (30.3, 38.1)	37.4 (33.0,43.1)	< 0.01
Fibrinogen(g/L)		3.3 (2.6, 4.2)	3.9 (2.9,4.9)	< 0.01
Sofa Score		0.0 (0.0,1.0)	1.0 (0.0,1.0)	< 0.01
NEWS Score		2.0 (0.0,5.0)	5.0 (2.0,8.0)	< 0.01
Death		98 (1.0%)	27 (7.1%)	< 0.01
High Risk		1,725 (17.5%)	154 (40.4%)	< 0.01

**Table 2:** Factors affecting hyperkalemia in patients admitted to the ER

Variables	Odds Ratio	Z	*p* > Z	95% CIs	
				Lower	Upper
Age	1.01	2.49	0.013	1.00	1.02
Gender	0.90	-0.80	0.425	0.69	1.17
Consciousness	0.89	-0.64	0.523	0.62	1.28
SBP(mmHg)	1.00	0.36	0.721	0.99	1.01
DBP(mmHg)	0.99	-1.22	0.222	0.98	1.00
Temperature(℃)	0.76	-2.22	0.026	0.59	0.97
Heart rates(beats/min)	1.00	-0.06	0.952	0.99	1.01
Respiratory rate(breaths/min)	1.00	0.25	0.803	0.98	1.03
Spo2	1.00	-1.11	0.266	0.99	1.00
NEWS score	1.05	1.55	0.121	0.99	1.12
Sodium(mmol/L)	0.83	-7.21	< 0.001	0.79	0.88
Calcium(mmol/L)	1.30	0.84	0.401	0.71	2.38
WBC(×10^9^/L)	0.96	-0.43	0.665	0.78	1.17
NEUT%	0.99	-0.36	0.722	0.95	1.04
Hematocrit	1.32	7.27	< 0.001	1.23	1.43
Hemoglobin (g/L)	0.92	-7.25	< 0.001	0.90	0.94
Glucose (mmol/L)	1.02	1.70	0.090	1.00	1.04
Creatinine(umol/L)	1.00	6.17	< 0.001	1.00	1.00
AST (U/L)	1.00	0.91	0.361	1.00	1.00
ALT (U/L)	1.00	0.28	0.777	1.00	1.00
Fibrinogen(g/L)	1.00	0.09	0.930	0.93	1.08
PT(secs.)	1.02	2.74	0.006	1.00	1.03
CO2CP(mmol/L)	0.91	-7.06	< 0.001	0.88	0.93
Albumin(g/L)	1.01	0.52	0.602	0.98	1.03
Osmolality(mmol/L)	1.01	1.88	0.060	1.00	1.03

### Severity and mortality of hyperkalemia patients

**Fig. 2:**
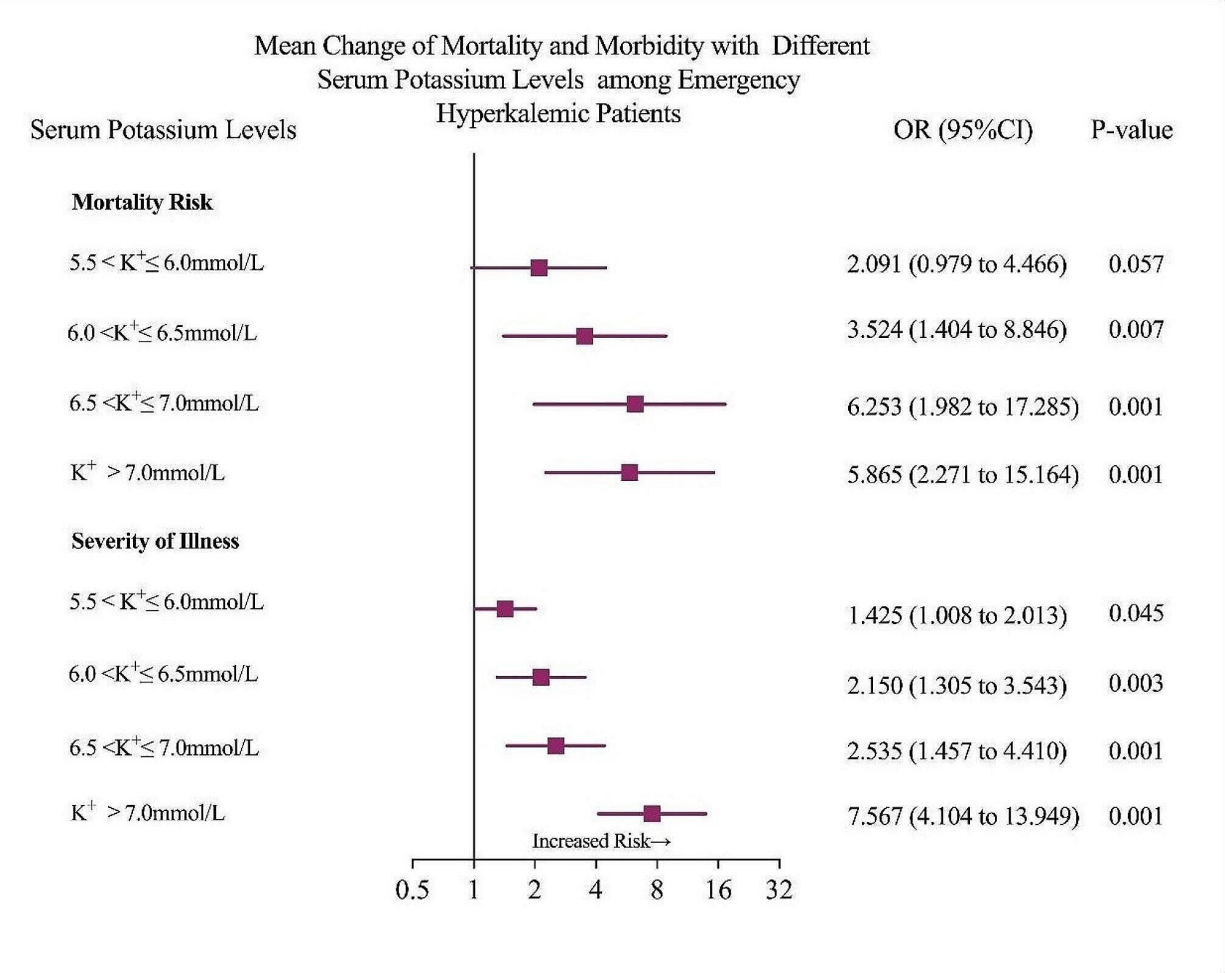
Mortality and morbidity of different serum potassium levels among emergency hyperkalemic patients

Of the 381 patients with hyperkalemia, 40.4% (*n* = 154) ones were categorized as critically ill (i.e., high risk) and 7.1% (*n* = 27) experienced in-hospital mortality. Following adjustment for patient confounding factors using the NEWS Score, an elevated potassium concentration was significantly associated with an increased odds ratio of death in the emergency room. Interestingly, patients with severe hyperkalemia (6.5mmol/L < [K^+^] ≤ 7mmol/L) exhibited the highest risk of death in the emergency room (OR = 6.253, 95%CI: 1.982–17.285, *p <* 0.001) when compared to patients with highly severe stage IV hyperkalemia ([K^+^] > 7mmol/L) (OR = 5.868, 95%CI: 2.271–15.164, *p <* 0.001). A similar trend is evident when assessing the requirement for high risk intensive care. The severity of hyperkalemia demonstrated a positively association with the severity of illness, with serum [K^+^] > 7mmol/L exhibiting the highest odds ratio for ICU admission (OR = 7.567, 95%CI: 4.104-13.949, *p =* 0.001). See Fig. 2 for further details.

**Fig. 3:**
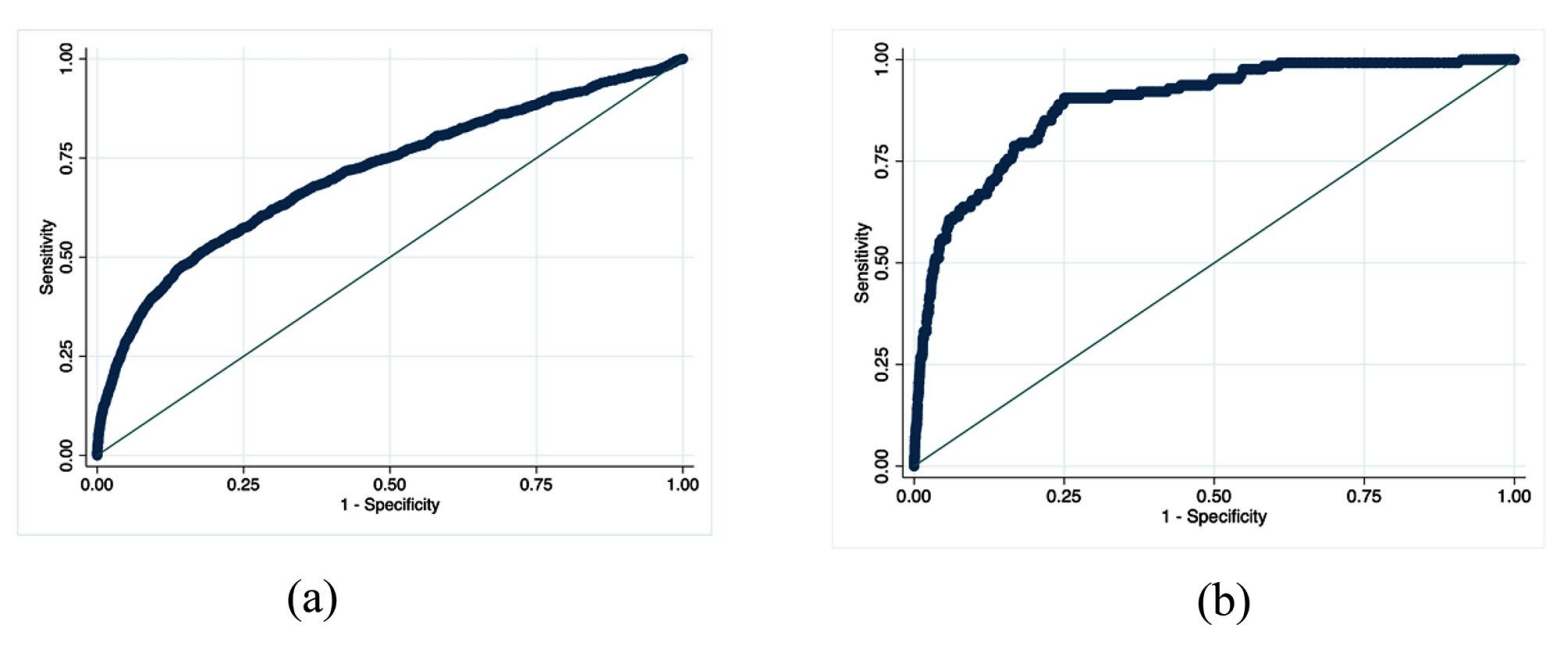
ROC analyses of (**a**) ICU admission and (**b**) in-room fatality risk

Our findings reveal that potassium concentration serves as a predictor for both mortality and illness severity among emergency room patients. The Area Under the Curve (AUC) of the ROC curve for forecasting the risk of mortality due to hyperkalemia in the emergency room is 0.890, while the AUC of the ROC curve for predicting the severity of the patient’s condition is 0.712. Both values suggest a relatively high predictive value (Fig. 3a and b).

### Efficacy of Potassium lowering interventions

**Fig. 4:**
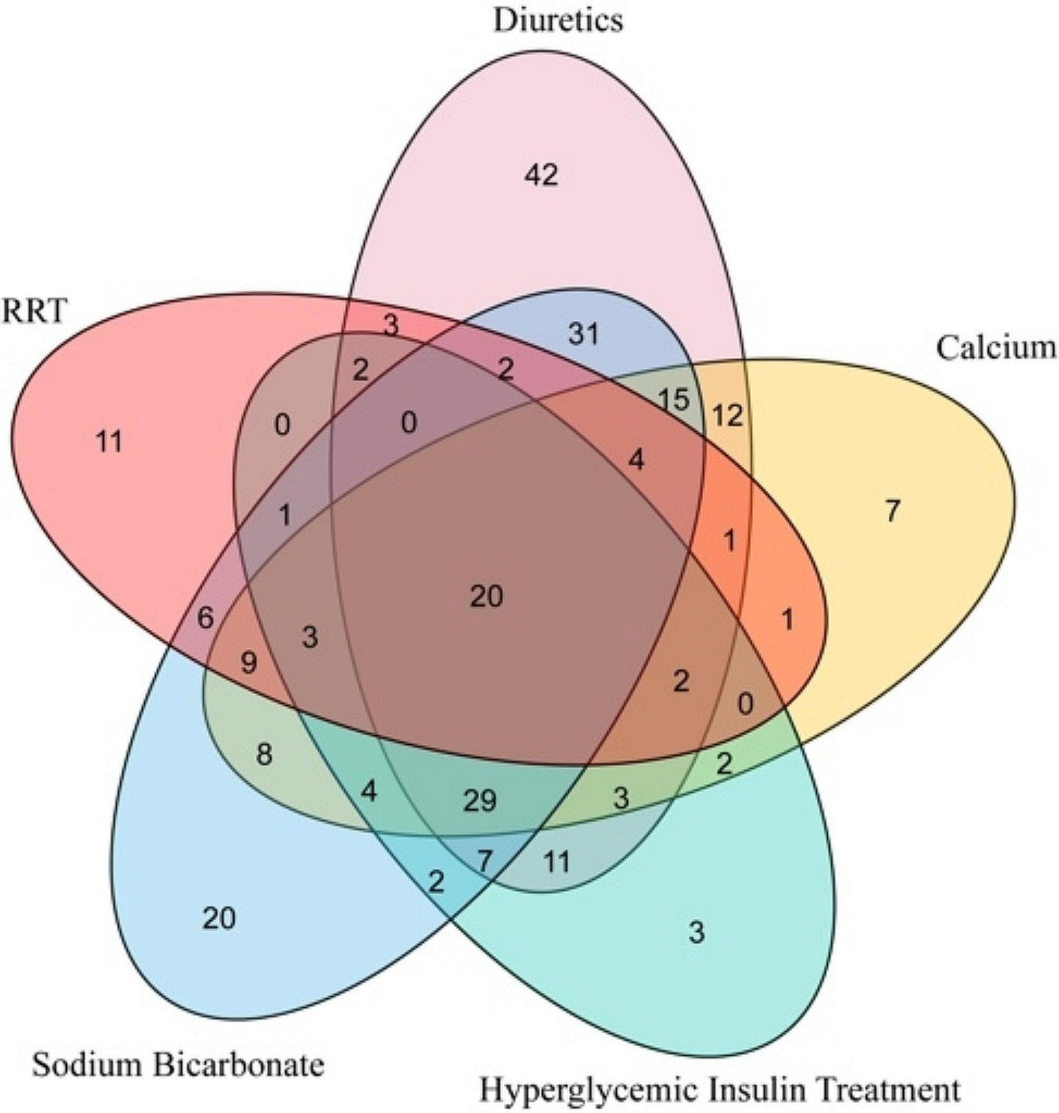
Venn diagram of therapeutic combinations with potassium-lowering drugs

In this study, we collected data from 328 emergency-admitted hyperkalemia patients who underwent various measures to reduce their potassium levels. Diuretic usage was the most common clinical measure to decrease potassium levels (57.32%, *n* = 188), followed by bicarbonate (50.91%, *n* = 167), calcium agents (37.2%, *n* = 122), insulin with glucose (27.74%, *n* = 91), and continuous renal replacement therapy (19.82%, *n* = 65). The relationship of combination therapy between potassium-lowering drugs is detailed in Fig. 4. After adjusting with the NEWS score (Table 3), renal replacement therapy therapy emerged as the most effective method for rapidly reducing potassium levels (OR = 1.90, 95%CI: 1.05–3.45, *p =* 0.035). Interestingly, the use of high glucose with insulin, bicarbonate, and calcium agents did not significantly impact the speed of potassium reduction. Notably, potassium levels were reduced more slowly with diuretics than without diuretics in this study. (OR = 0.33, 95%CI: 0.20–0.55, *p =* 0.001).

**Table 3:** Effect Of potassium reduction interventions

Rate of potassium Reduction($\varDelta k/k$1)	Univariable			Multivariable		
	OR	95%CI	*p*	OR	95%CI	*p*
Diuretic	0.38	0.24–0.61	< 0.001	0.33	0.20–0.55	0.001
Calcium (Chemistry)	1.21	0.77–1.90	0.419	1.12	0.63–1.97	0.704
Hyperglycemic insulin	1.02	0.63–1.67	0.923	1.16	0.64–2.10	0.623
Sodium bicarbonate	1.38	0.89–2.15	0.147	1.50	0.90–2.51	0.123
Renal replacement therapy	2.16	1.22–3.83	0.008	1.90	1.05–3.45	0.035

## Discussion

This study provides a thorough epidemiological analysis of hyperkalemia among patients in emergency rooms and several noteworthy findings emerged. Firstly, elevated potassium is not uncommon, with an incidence rate of 2.98%. In addition, elevated potassium levels significantly increase the risk of mortality, and is a strong indicator of disease severity and death in emergency rooms (AUC 0.89 for mortality and 0.71 for ICU admission). Notably, patients diagnosed with Stage III hyperkalemia (Severe hyperkalemia,6.5< [K^+^] ≤ 7mmol/L) in the emergency exhibited the highest risk of mortality more than six-fold compared to patients with normal K^+^, surpassing even Stage IV hyperkalemia. This finding suggests the possibility of delayed recognition among individuals with moderately elevated potassium concentrations. CRRT emerged as the most effective intervention for high potassium levels. However, despite their frequent use, diuretics appear less effective at reducing blood potassium compared to the other four methods studied here. This study contributes to a deeper understanding of the associated risk of hyperkalemia in clinical settings and offer evidence to support decisions regarding potassium-lowing interventions.

The reported incidence of hyperkalemia varies across different studies. Humphrey et al. reported a global prevalence of hyperkalemia at 6.3% [13]. A study of U.S emergency departments reported an incidence rate of 3.6% [14–17], whereas Canadian emergency department reported a rate of 2.6% [18]. In a multicenter study conducted by Xu et al., the incidence rate in Chinese emergency departments was reported to be 3.52% [19]. The rate in this study was higher than that of the Canadian study but slightly lower than that of the United States at 2.98%, suggesting our findings are within a normal range. Additionally, the observed variation in incidence could be attributed to diverse populations included in the different studies. For instance, in the Xu et al. study participants were predominantly hypertensive, with dyslipidemia and having other trauma [17]. While those in the Canadian study were mainly patients with coronary artery disease and heart failure [18]. Past studies in China have primarily enrolled patients with heart failure, diabetes, hypertension, and chronic kidney disease. This study primarily included individuals presenting with complaints of disturbances in consciousness and with cardiovascular symptoms. Prior investigations have indicated the presence of cardiovascular symptoms and disordered consciousness can increase the likelihood of developing hyperkalemia by approximately 40% [20]. Taken together, these findings provide only partial insight into regional variations in the incidence of hyperkalemia. However, this study highlights the importance of promptly assessing serum potassium levels for emergency department patients presenting with disturbances of consciousness and symptoms of cardiovascular disease.

This study revealed that patients at Stage III (6.5 < [K^+^] ≤ 7mmol/L) had the highest risk of death in the emergency room, although the probability of being admitted to an intensive care unit was lower compared to those with Stage IV hyperkalemia ([K^+^] > 7mmol/L). These findings strongly suggest a need for increase our attention on individuals with extremely high potassium levels, but also that patients with potassium levels within the range of 6.5-7.0 may not receive adequate attention. This is particularly common in the ER, where medical resources are relatively limited. Our team’s earlier work demonstrated that ERs in developing countries, such as China, often encounter an excess of patients who do not actually require emergency services. This situation influences ‘patient encounter time’ and therefore individuals with moderately elevated potassium may not be monitored as they should. Given that hyperkalemia can lead to serious arrhythmias and life-threatening risks [21, 22], our study aligns with a retrospective study in 2017 which found a 5-fold increase in the probability of death compared to patients with ‘normal’ potassium levels (3.5-5.0mmol/L) [17]. This provides further context which must be considered in further detail, and it may be necessary to reconsider the categorization ‘moderate’ and conduct further research into the implications for triaging.

Another notable finding in our study that merits special attention is the frequent use of diuretics to lower potassium levels (*n* = 188, 57.32%). However, the speed of potassium reduction with diuretics was slower than in those who did not receive this intervention. These findings are consistent with several other studies [15, 23], indicating that although diuretics can effectively enhance the excretion of potassium, their role in the treatment of acute hyperkalemia remains unclear, and should only be considered as an adjunctive treatment. Several factors may contribute to these findings, particularly among ER patients. Firstly, patients with hyperkalemia included in this study often presented with renal insufficiency, limiting the effectiveness of diuretics and also because fluid status is often inadequate in emergency admission patients. The inappropriate use of diuretics may further worsen fluid status, slowing down the reduction of serum potassium. Lastly, high doses of diuretics may cause renal damage [24], reducing the efficiency of renal reabsorption of both water and sodium, thereby limiting the excretion of potassium. This has significant implications, suggesting that clinicians need to carefully assess the patient’s condition and renal function during the treatment of hyperkalemia. In case of severe hyperkalemia requiring prompt reduction of serum potassium, diuretics may not be the optimal choice. In our study, up to 20% of patients received renal replacement therapy treatment which emerged as the most efficient method for reducing potassium. Numerous studies have shown that compared to other potassium-lowering measures, renal replacement therapy remains the most effective approach to reduce serum potassium [15, 25]. Nevertheless, a nationwide study in China revealed that only 61.46% of emergency departments had access to renal replacement therapy [26]. Suggesting potential limitations in its use due to inadequate manpower, equipment, and relatively high costs.

The observed higher mortality in the group of patients with potassium levels between 6.5 and 7.0 may be partially explained by delayed admission to the intensive care unit and a lower rate of receiving Continuous Renal Replacement Therapy. This raises important questions regarding socio-economic factors, from both the patients’ and practitioners’ perspectives. For instance, several studies have highlighted income and economic stability, education level and social support networks as key factors in health-seeking behaviours [27, 28], as are cultural and social norms. This in turn implicates access disparities, health care insurance and geographical healthcare imbalances as reported by Jin et al., the allocation of resources for emergency medical services in China exhibits geographical disparities, with pronounced inequalities between secondary and tertiary hospitals, a disparity that is notably accentuated in rural regions [29]. There are also other issues, from a practitioners’ perspective, such as the perceived frailty of patients and perhaps the clinicians’ reliance on chronological age and the sensitivity to what might be perceived as ‘alarm symptoms.’ Along with the socio-economic disparities which likely influence health-seeking behaviours these issues require further research to understand delayed admissions and the factors which influence clinical judgments in relation to the decision to administer CRRT. This might include electrolyte imbalance, hemodynamic stability, underlying conditions, and perceived urgency as well as responses to other therapies. This requires further primary qualitative research to explore decision making processes in situ.

## Limitations

This study had several limitations which should be addressed before reaching conclusions. Firstly, this was an observational study which pioneers the use of a large database of emergency admission patients for such research in China; however, the sample size of hyperkalemia patients was relatively small. Additionally, the inability to collect extensive data, including comorbidities, renal function, and long-term mortality among others, limited our ability to analyze the etiologies of elevated serum potassium. Also, the data used in this study was from only two regional medical centers of large tertiary hospitals, and the results may not be fully representative of practices across diverse healthcare settings. For instance, emergency renal replacement therapy may not be available in some suburban hospitals. Thirdly, when evaluating the effect of different therapies, despite adjusting for possible biases, our data did not support the analysis of the effects of each individual therapy as well as those therapies that were not available in our study such as intestinal potassium exchange resins. Therefore, caution is necessary when applying this subset of results in clinical practices. Finally, considering the diverse pathophysiological mechanisms underlying hyperkalemia, including factors such as renal failure, drug interactions, and imbalances between intracellular and extracellular potassium, further research is recommended within these specific subgroups and perhaps through a 360-degree analysis of the pathways that balance the kidney and the heart physiologies.

## Conclusion

Hyperkalaemia is not uncommon in emergency room patients and is associated with increased mortality and the need for ICU admission. Diuretics should be used with caution and CRRT remained the most effective treatment. However, there are disparities with some high-risk patients not receiving CRRT interventions. Factors which influence health-seeking behaviours are not well-understood in this field nor are the factors which influence clinical judgments in ERs, these are two areas which require further investigation to improve outcomes.

## Data Availability

The data that support the findings of this study are not openly available due to reasons of sensitivity and are available from the corresponding author upon reasonable request.

## Abbreviations

CETAT: Chinese Emergency Triage Assessment and Treatment database
ER: Emergency Room
ROC: Receiver Operating Characteristic
AUC: Area Under the Curve, IV: Intravenoustherapy
USTC: University of Science and Technology of China
ICU: Intensive Care Unit
SBP: Systolic Blood Pressure
DBP: Diastolic Blood Pressure
WBC: White Blood Cell Count NEUT%:Neutrophil Percentage
PLT: Platelet Count
ALT: Alanine Aminotransferas
AST: Aspartate Aminotransferase
TBIL: Total Bilirubin
CO2CP: Carbon Dioxide Combining Power
PT: Prothrombin Time
Sofa Score: Sequential Organ Failure Assessment
NEWS Score: National Early Warning Score IQR: Interquartile Range
OR: Odds Ratio
CRRT: Continuous Renal Replacement Therapy
K: Kalium
CI: Confidence Interva
